# Hydatidose péricardique

**DOI:** 10.11604/pamj.2015.20.375.3124

**Published:** 2015-04-16

**Authors:** Hicham Fenane, El mehdi Maidi, Mohamed Bouchikh, Damsane Lamboni, Abdellah Achir, Fahd Ouchen, Mbola Oyali, Mohamed Caidi, Said Al Aziz, Abdellatif Benosman

**Affiliations:** 1 Service de Chirurgie Thoracique, Hôpital Ibn Sina, Rabat, Maroc

**Keywords:** Hydatidose péricardique, atteinte cardiaque, Maghreb, cardiac hydatidosis, cardiac involvement, Maghreb

## Abstract

L'hydatidose est un problème de santé publique dans les pays d'endémie dont le Maghreb. L'atteinte péricardique reste rare, le diagnostic est parfois tardif à cause de la non spécificité des symptômes, et repose essentiellement sur les données de l'imagerie. Nous rapportons deux observations d'hydatidose péricardique sans atteinte cardiaque.

## Introduction

L'hydatidose péricardique sans atteinte cardiaque est extrêmement rare même en pays d'endémies, elle est due au développement de la forme larvaire du ténia d’*Echinococcus granulosus*. Elle se manifeste habituellement par une dyspnée et une altération de l’état général. Le diagnostic repose sur les données de l'imagerie. Et le traitement est chirurgical. Nous rapportant deux observations cliniques de deux patientes opérés pour hydatidose péricardique.

## Patient et observation

### Observation 1

**Figure 1:**
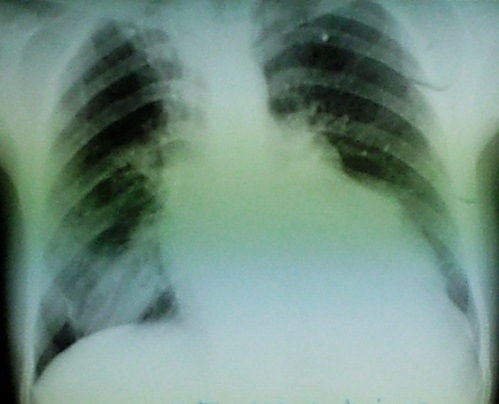
Radiographie thoracique montrant une cardiomégalie avec un bord gauche polylobé et une opacité basale droite

**Figure 2:**
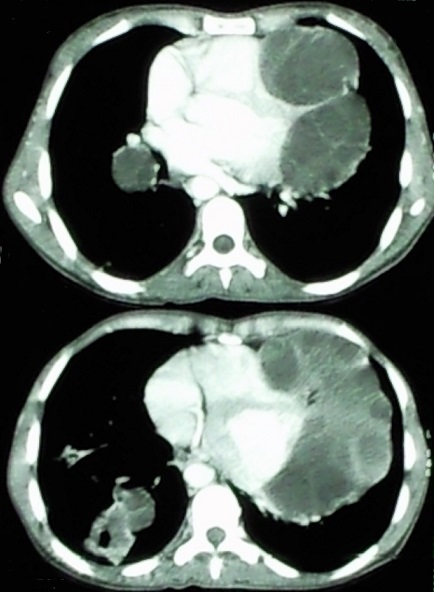
TDM thoracique montrant des lésions kystiques multi-vésiculaires du bord gauche du cœur, et des formations kystiques pulmonaires droites

**Figure 3:**
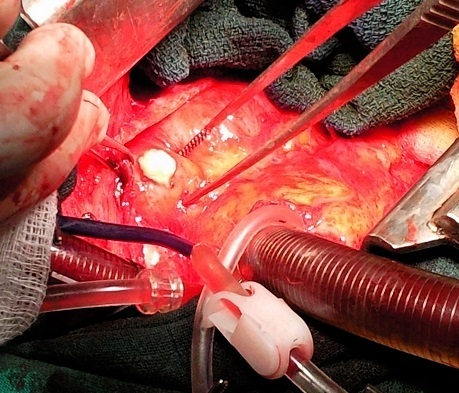
Vue opératoire montrant l'extraction de la membrane après installation de la circulation extra-corporelle

Patiente de 40 ans, avec notion de contact avec les chiens qui était retrouvée dans les antécédents, et qui présentait depuis une année une dyspnée d'effort d'aggravation progressive avec des douleurs thoraciques. L'examen clinique retrouvait un assourdissement des bruits cardiaques, L'ECG avait montré un microvoltage. La patiente avait bénéficié d'un bilan radiologique objectivant sur la radiographie du thorax (Figure 1) une cardiomégalie avec une opacité bilobée au niveau du bord gauche du cœur, la Tomodensitométrie (TDM) (Figure 2) avait montré de Multiples lésions kystiques renfermant de multiples images vésiculaires contigües en para-cardiaque gauche arrivant à la hauteur du tronc de l'artère pulmonaire et exerçant un effet de masse sur le parenchyme pulmonaire. La TDM retrouvait également deux lésions kystiques du parenchyme pulmonaire droit évoquant des kystes hydatiques pulmonaire associés surinfectés. L’échocardiographie avait montré la présence de multiples formations kystiques péricardiques et ayant des rapports étroits avec le massif cardiaque, et qui avaient un contact avec le tronc de l'artère pulmonaire commune. L’échographie abdominale était sans anomalies, Le bilan biologique trouvait une hyperleucocytose 10500 blanc à prédominance d’éosinophile, la sérologie hydatique était négative. La patiente avait été mise sous traitement médical Albendazole 15mg/Kg durant un mois en préopératoire. La patiente été opéré par stérnotomie et sous circulation extracorporelle (Figure 3). Avant ouverture du péricarde le champ opératoire était entouré de sérum salé hypertonique, le liquide intrakystique était aspiré à la seringue. On avait enlevé la membrane germinale ainsi que des vésicules filles. Les suites opératoires étaient simples. Et la patiente fut adressée en chirurgie thoracique pour cure des kystes pulmonaires.

### Observation 2

**Figure 4:**
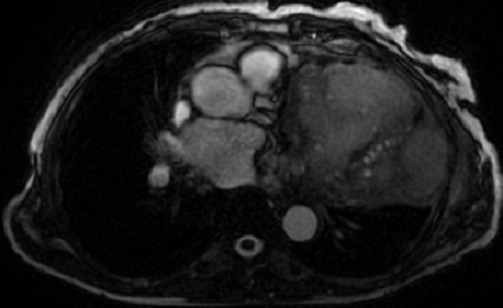
IRM montrant une formation kystique paracardiaque gauche en hyper signal en T2

Patiente de 66 ans qui vivait en milieu rural et qui avait un contact avec les chiens. Qui présentait depuis 8 mois une dyspnée stade II et une toux sèche, l’évolution c’était faite dans un contexte d'apyrexie et de conservation de l’état général. L'examen clinique montrait un assourdissement des bruits cardiaques, le reste de l'examen clinique était sans anomalies. L'ECG montrait un microvoltage. La patiente avait bénéficié d'un bilan radiologique qui montrait sur la radiographie du thorax une cardiomégalie. Une échocardiographie avait montré une masse kystique en rapport avec la pointe et la paroi latérale du ventricule gauche. la TDM avait montré une grosse masse kystique multi vésiculaire paracardiaque gauche en intrapéricardique, l'imagerie par résonnance magnétique IRM (Figure 4) avait confirmé l'origine péricardique de la lésion qui était en hyposignal en T1, hypersignal en T2, et avait confirmé la localisation à gauche de toutes les lésions. L’échographie abdominale était normale. La sérologie hydatique était positive, le reste du bilan biologique était normal. La patiente avait été opérée par stérnotomie médiane permettant l'extraction de membrane et la réalisation d'une toilette péricardique au sérum salé hypertonique. Les suites opératoires étaient simples. La patiente était sortie sous traitement médical: Albendazole 15mg/Kg.

## Discussion

L'hydatidose péricardique est une maladie rare, elle représente 0,2 à 2% des cas d'hydatidose [1, 2]. L'atteinte cardiaque est isolée dans 1/3 des cas, dans les 2/3 des cas elle est associée à une atteinte pulmonaire, hépatique ou médiastinale [1, 3]. Le patient est contaminé soit indirectement en ingérant l'eau ou les aliments contaminés par les œufs du parasite soit directement à travers le contact avec les chiens. La larve d’*E. granulosus* arrive dans les cavités cardiaques gauches après avoir échappé au filtre hépatique, atteint l'oreillette droite et de là le cœur gauche par la circulation pulmonaire, voire un foramen ovale perméable [4]. À partir du ventricule gauche, les larves sont expulsées dans la grande circulation et par le biais des artères coronaires, le parasite envahit le myocarde [5]. Toutes les localisations du kyste hydatique du c'ur sont possibles. Le parasite siège par fréquence dans le ventricule gauche dans 50 à 60% des cas, le septum interventriculaire dans 10-20% des cas, le ventricule droit dans 5-15% des cas, la localisation cardio-péricardique constitue 10-15% des cas. La localisation péricardique sans atteinte cardiaque est donc extrêmement rare [1, 2]. Et semble toujours secondaire. Les symptômes des kystes hydatiques péricardiques sont non spécifiques et variées, en rapport avec le nombre la taille, et le siège des kystes. Ils sont généralement dus à la pression externe exercée par l'augmentation de la taille du kyste hydatique sur le myocarde, la rupture du kyste dans la cavité péricardique peut être responsable d'un épanchement aigue avec tableau de péricardite aigue sérofibrineuse ou purulente et dont l’évolution est soit vers la tamponnade ou la constriction [1, 6], des formes asymptomatiques ont été rapportées [2, 3]. La sérologie hydatique n'est positive que dans la moitié des cas des kystes hydatiques du cœur, une de nos patientes avait une sérologie positive. La méthode d'Elisa et l'immunofluorescence indirecte sont les tests les plus sensibles. L'immunoélectrophorèse est le test le plus spécifique [1, 2, 4]. La radiographie du thorax est peu contributive. Elle peut être normale en cas de kyste de petite taille ou à développement intra cavitaire. Et montre soit une déformation cardiaque uni ou bilobée dans la moitié des cas [1, 2] soit des calcifications arciformes ou en plaque observées dans un tiers des cas [1], soit une éventuelle localisation pulmonaire associée [1]. Le diagnostic est basé sur les techniques d'imagerie cardiaque, et la sérologie hydatique. L’échocardiographie transthoracique bidimensionnelle constitue actuellement l'examen de choix pour le diagnostic des kystes hydatiques cardio-péricardiques [2, 7]. Elle montre une formation anéchogène à paroi fine avec décollement de membrane ou aspect multi-vésiculaire qui sont hautement évocateurs de l'origine hydatique. Elle permet de préciser la localisation, les rapports de la lésion kystique, la présence d'un épanchement péricardique ou pleural associé. Chez nos patientes elle avait permit de localiser les kystes et de préciser leur rapports anatomiques. La tomodensitomètrie TDM et l'imagerie par résonance magnétique IRM s'avèrent nécessaire soit pour écarter les autres diagnostics de masse kystique, soit pour en préciser les rapports. Sur la TDM le kyste hydatique se présente comme une formation arrondie, hypo-dense homogène, uniloculaire à paroi fine le plus souvent, non rehaussée par le produit de contraste. La mise en évidence de calcifications pariétales en TDM est en faveur du diagnostic d'hydatidose. L'IRM montre le kyste en hypo-signal T1 et en hyper-signal T2 elle permet de voir avec précision la topographie des kystes et leurs rapports anatomiques et apporte ainsi une aide importante au chirurgien pour guider le geste chirurgical [1], c’était le cas de nos patientes où elle avait précisé le siège des kystes et leurs rapports.

Le traitement des kystes hydatiques du péricarde est chirurgical. Il consiste en une excision des kystes pour éviter les complications qui peuvent être mortels en cas de rupture, même quand il s'agit de patients asymptomatiques [2, 8]. Le traitement médical représente le traitement de choix pour les patients non opérables du fait de kystes hydatiques trop nombreux, ou de terrain débilité, ou un traitement complémentaire d'une intervention chirurgicale lorsqu'il y a risque de dissémination. Le produit le plus utilisé est l'Albendazole^®^ à la dose de 10 à 15 mg/kg par jour par cures d'un mois espacées de 15 jours pendant six mois [2].

## Conclusion

La localisation péricardique de l'hydatidose est rare, la symptomatologie n'est pas spécifique et est parfois tardive. Le diagnostic est basé sur les données de l'imagerie, et le traitement est chirurgical.
